# Integration of Transcriptomics and Non-Targeted Metabolomics Reveals the Underlying Mechanism of Skeletal Muscle Development in Duck during Embryonic Stage

**DOI:** 10.3390/ijms24065214

**Published:** 2023-03-08

**Authors:** Zhigang Hu, Xiaolin Liu

**Affiliations:** College of Animal Science and Technology, Northwest A&F University, Xianyang 712100, China

**Keywords:** Pekin duck, skeletal muscle, transcriptome, metabolome, pathway

## Abstract

Skeletal muscle is an important economic trait in duck breeding; however, little is known about the molecular mechanisms of its embryonic development. Here, the transcriptomes and metabolomes of breast muscle of Pekin duck from 15 (E15_BM), 21 (E21_BM), and 27 (E27_BM) days of incubation were compared and analyzed. The metabolome results showed that the differentially accumulated metabolites (DAMs), including the up-regulated metabolites, l-glutamic acid, n-acetyl-1-aspartylglutamic acid, l-2-aminoadipic acid, 3-hydroxybutyric acid, bilirubin, and the significantly down-regulated metabolites, palmitic acid, 4-guanidinobutanoate, myristic acid, 3-dehydroxycarnitine, and s-adenosylmethioninamine, were mainly enriched in metabolic pathways, biosynthesis of secondary metabolites, biosynthesis of cofactors, protein digestion and absorption, and histidine metabolism, suggesting that these pathways may play important roles in the muscle development of duck during the embryonic stage. Moreover, a total of 2142 (1552 up-regulated and 590 down-regulated), 4873 (3810 up-regulated and 1063 down-regulated), and 2401 (1606 up-regulated and 795 down-regulated) DEGs were identified from E15_BM vs. E21_BM, E15_BM vs. E27_BM and E21_BM vs. E27_BM in the transcriptome, respectively. The significantly enriched GO terms from biological processes were positive regulation of cell proliferation, regulation of cell cycle, actin filament organization, and regulation of actin cytoskeleton organization, which were associated with muscle or cell growth and development. Seven significant pathways, highly enriched by *FYN*, *PTK2*, *PXN*, *CRK*, *CRKL*, *PAK*, *RHOA*, *ROCK*, *INSR*, *PDPK1*, and *ARHGEF*, were focal adhesion, regulation of actin cytoskeleton, wnt signaling pathway, insulin signaling pathway, extracellular matrix (ECM)-receptor interaction, cell cycle, and adherens junction, which participated in regulating the development of skeletal muscle in Pekin duck during the embryonic stage. KEGG pathway analysis of the integrated transcriptome and metabolome indicated that the pathways, including arginine and proline metabolism, protein digestion and absorption, and histidine metabolism, were involved in regulating skeletal muscle development in embryonic Pekin duck. These findings suggested that the candidate genes and metabolites involved in crucial biological pathways may regulate muscle development in the Pekin duck at the embryonic stage, and increased our understanding of the molecular mechanisms underlying the avian muscle development.

## 1. Introduction

Skeletal muscle is mainly composed of muscle cells, which performs functions through contraction and relaxation. Muscle growth and development, regulated by transcription and post-transcription and pathways, is a complex process [1]. Skeletal muscle development can be divided into three stages. The first stage is the formation of muscle fibers in the embryonic stage, including (1) the proliferation and differentiation of myoblast precursors, (2) the fusion of myoblasts into multinucleated myotubes, and (3) the formation of mature muscle fibers. The second stage is the development of myofibers in the fetus, which is mainly the change in various fiber types. The third stage is muscle regeneration in adulthood, mainly the increase in myofiber diameter and length and the repair of damaged muscle fibers [2,3]. Among them, the first stage is very important as the result of the embryonic stage determines the number of muscle fibers [4]. Skeletal muscle synthesis in avian embryos can be divided into two processes: primary and secondary muscle fiber formation. A small number of embryonic myoblasts begin to fuse, and the first multinucleated myofiber in vertebrate embryos is formed. Once the scaffold of primary myofibers is established, secondary myofibers can be formed through the fusion between embryonic myoblasts or with primary muscle fibers. Generally, the primary muscle fibers are similar to the slow muscle fibers of adult animals, while the secondary muscle fibers show the characteristics of fast muscle fibers [5]. Some studies have shown that the primary muscle fibers of chicken form from about E6 (the 6th day of incubation), and the secondary myofibers begin to differentiate from E12 to E16 (the 12th to 16th day of incubation) [6]. The difference in myogenesis between mammalian and avian embryos is that the development of avian embryos does not depend on the maternal uterus, but only on the nutrition provided by the yolk. Therefore, it is easy and meaningful to study the differences in regulatory genes and metabolites of skeletal muscle development in birds during different incubation periods.

Transcriptomics is a method to study gene expression and transcriptional regulation at the RNA level of specific cell types, tissues, or organisms [7]. In recent years, RNA-Seq has been widely used in the study of animal transcriptome [8,9]. Compared with other gene expression profiling methods, RNA-Seq has the advantages in detecting mRNA expression in different tissues or at different stages of development, helping to reveal new genes, splice variants, and regulatory pathways [10,11,12,13]. Various metabolites and related metabolic pathways in the complex regulatory network were identified by detecting endogenous low-molecular-weight metabolites (molecular weight within 1000 Da) in cells, tissues, organs, or organisms before and after treatment using liquid chromatography/mass spectrometry (LC-MS), gas chromatography/mass spectrometry (GC-MS), and nuclear magnetic resonance (NMR). Metabolomics data identify changes in bioactive compounds during animal development, while RNA-seq data identify genes that regulate metabolic changes. Spurred by a massive advance in technology, the molecular mechanisms of organisms (genome, transcriptome, proteome, and metabolome) were systematically characterized quantitatively by multi-omics analysis [14]. The metabonomics analysis results provide different metabolites, which can make up for the lack of data in transcriptomics analysis, and the life activities and molecular regulation mechanisms can be explored from a multi-level perspective through the combined analysis of transcriptomics and metabonomics [15].

To date, there have been few studies to explore the potential key factors involved in the skeletal muscle development of embryonic duck using transcriptomics and metabolomics techniques. Our previous research carried out transcriptome sequencing on the skeletal muscle of Pekin duck in the embryonic period. The differentially expressed genes such as *MYL4* and *IGF2BP1* and the regulatory pathways such as focal adhesion and ECM-receptor interaction play a crucial role in the development of duck muscle [16]. Liu et al. found that the metabolism of duck meat changes with age (27, 50, 170, and 500 days of age) by NMR, that is, lactate and anserine increased with age, fumarate, betaine, taurine, inosine, and alkyl-substituted free amino acids decreased [17]. Zhou et al. conducted a comparative analysis of multi-omics data (genome, transcriptome, and metabolome) from the breast meat of Lianchen white ducks (LW) and Mianyang Shellducks (MS) at 300 days of age. The results showed that LW ducks had unique breed-specific genetic characteristics, including many differentially expressed mutant genes related to amino acid metabolism and transport activities. Moreover, the concentration of L-arginine, L-ornithine, and L-lysine in LW duck meat was significantly higher than that in MS duck muscle. In addition, guanosine monophosphate (GMP) was significantly higher in LW muscle, while L-aspartic acid was significantly enriched in MS duck meat [18]. A combination of gene function and metabolites are useful for the comprehensive study of the developmental mechanism of Pekin duck skeletal muscle during the embryonic stage. In this study, the dynamic metabolomic and transcriptomic profiles from breast muscle of Pekin duck at embryonic days 15, 21, and 27 were comparatively investigated, and some potential metabolites and the corresponding differentially expressed genes at the molecular levels were identified. This study revealed crucial metabolic pathways and metabolites of muscle development in Pekin duck at embryonic stages, providing important insights into the mechanisms underlying the muscle development in avian.

## 2. Results

### 2.1. Metabolome Analysis

#### 2.1.1. Overview of the Metabolomic Profiling

**Figure 1:**
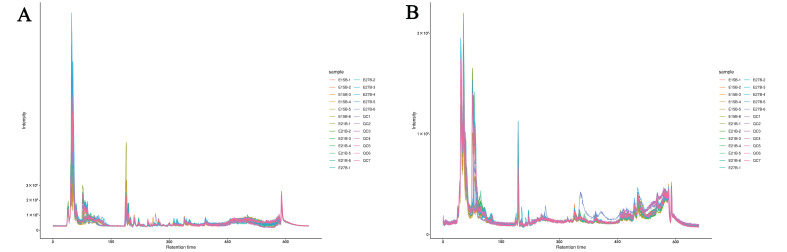
Overlapping analysis of total ion chromatogram. (**A**) The electrospray ionization positive ion mode (pos); (**B**) the electrospray ionization negative ion mode (neg).

During LC-MS/MS analysis, one quality control (QC) sample was injected into every 10 samples to monitor the repeatability of the analysis process. Therefore, the repeatability of metabolite extraction and detection can be judged by overlapping analysis of total ion chromatogram (TIC) in different QC samples. The TIC results show that the retention time and peak intensities were consistent, which indicated that the signal was stable when the same sample was detected at different times (Figure 1). 

**Figure 2:**
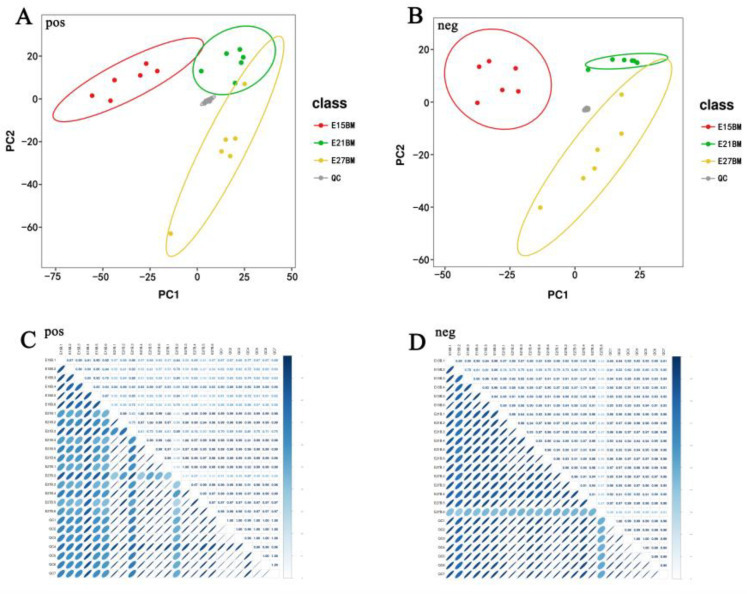
PCA analysis and Pearson’s correlation coefficients among samples. (**A**,**B**) PCA analysis; (**C**,**D**) Pearson’s correlation coefficients. Note: E15_BM represents breast muscle of Pekin duck on day 15 of the incubation period. The same is shown below.

In this study, the sample metabolites were analyzed by PCA to evaluate the reliability of the data, and preliminarily understand the overall metabolic difference and the degree of variation between the samples in each group. PCA analysis of samples from each group showed an obvious separation and formed a cluster, indicating that there are metabolic markers that can be classified (Figure 2A,B). Moreover, the results of Pearson correlation coefficient confirmed the high repeatability of samples within the group and ensured the reliability of screening for differential metabolites (Figure 2C,D).

The overall differences in metabolites in duck muscle samples at three time points were observed by using partial least squares discriminant analyses (PLS-DA), and the three comparison groups have better separation in positive and negative ion modes (Figure S1). The PLS-DA model of each comparison group was established and then evaluated. R2 (cum) represents the interpretation ability of the model and Q2 (cum) represents the prediction ability of the model. When R2Y (cum) > 0.50 and Q2 (cum) > 0.50, it indicates that the model has high stability, good interpretation and prediction ability. Then, a permutation test of the PLS-DA model was performed (200 test times), and Q2 < 0 indicated that the model did not have overfitting. In this study, Q2 was −0.08 and −0.02, respectively, indicating that differential metabolites can be screened and analyzed according to the model (Figure S2).

#### 2.1.2. Identification of Differentially Accumulated Metabolites

**Figure 3:**
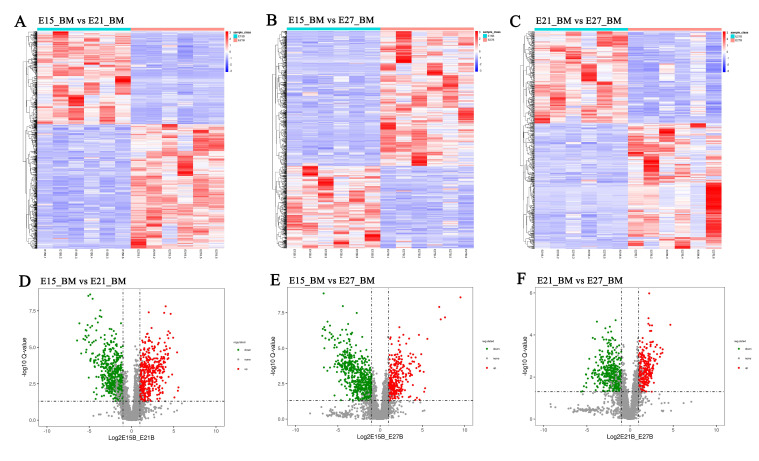
Identification of differentially accumulated metabolites among three comparison groups. (**A**–**C**) Hierarchical cluster analysis; (**D**–**F**) Volcano plot of DAMs.

**Figure 4:**
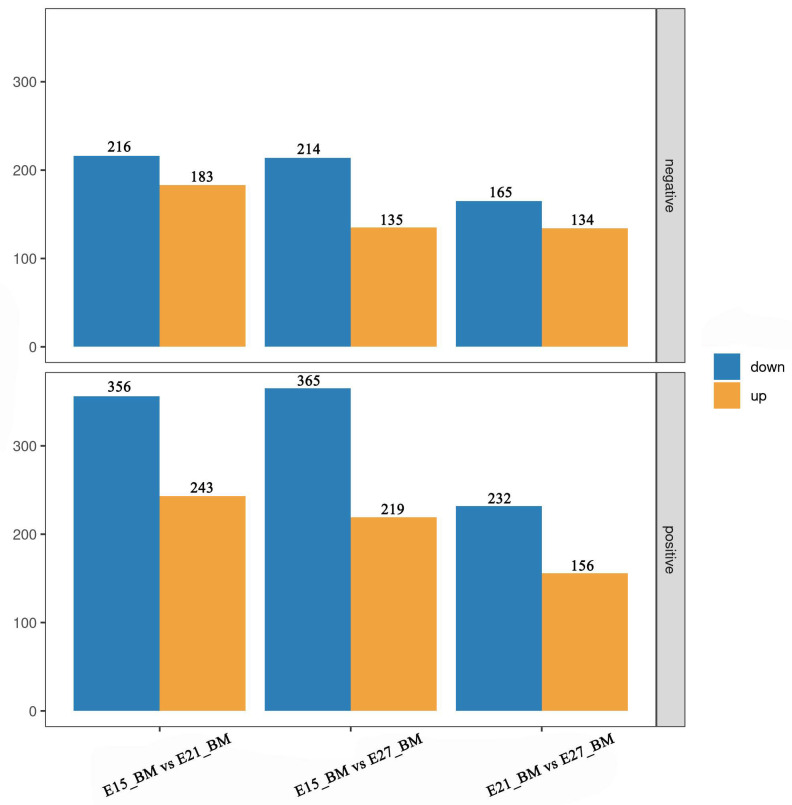
Number of differentially accumulated metabolites (DAMs) among three comparison groups.

**Figure 5:**
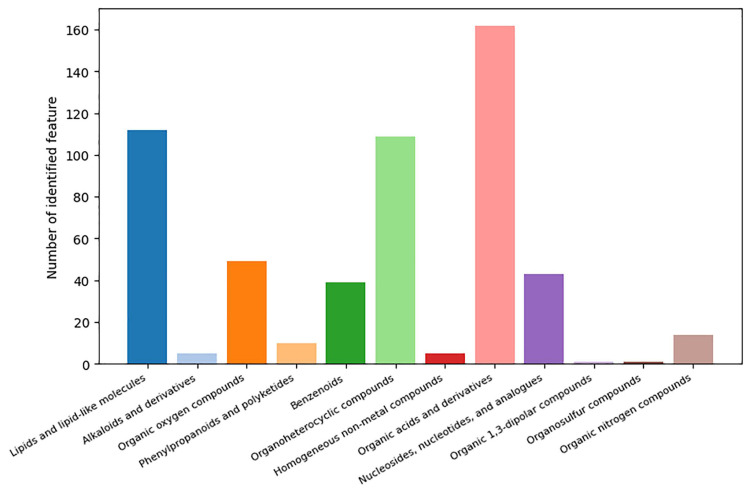
Component analysis of the identified metabolites based on HMDB database.

After qualitative and quantitative analysis of the detected metabolites, differentially accumulated metabolites were found in each group based on fold change ≥2 or ≤1/2, *p* value < 0.05 and VIP ≥ 1. Hierarchical cluster analysis was performed to evaluate the DAM accumulation patterns (Figure 3A–C). There were 599, 584, 388 and 399, 349, 299 DAMs were identified in positive ion mode (pos) and negative ion mode (neg) of three comparison groups, respectively (Figure 3D–F, Table S1). For E15_BM vs. E21_BM, 243 (pos) and 183 (neg) were up-regulated, and 356 (pos) and 216 (neg) were down-regulated. Of the DAMs identified between E15_BM and E27_BM, 219 (pos) and 135 (neg), and 365 (pos) and 214 (neg) metabolites were up-regulated and down-regulated, respectively. Of the number of metabolites differentially accumulated in E21_BM compared to E27_BM, 156 (pos) and 134 (neg) metabolites were upregulated, and 232 (pos) and 165 (neg) metabolites were downregulated, respectively (Figure 4). The top 10 up- and down-regulated DAMs from three comparison groups were shown in Table S2. Of all DAMs, “Organic acids and derivatives”, “Lipids and lipid-like molecules”, “Organoheterocyclic compound”, “Organic oxygen compounds”, “Nucleosides, nucleotides, and analogues”, and “Benzenoids” accounted for a large proportion based on the HMDB database (Figure 5).

#### 2.1.3. KEGG Analysis of Differentially Accumulated Metabolites

**Figure 6:**
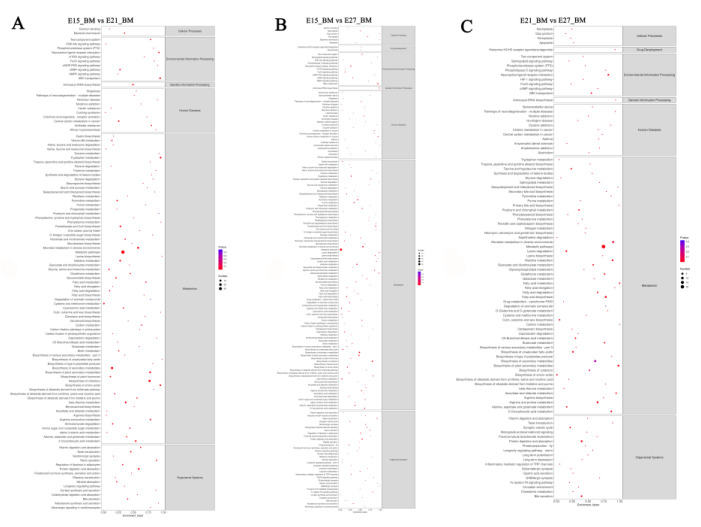
KEGG pathways of differentially accumulated metabolites (DAMs) enrichment in breast muscle. (**A**) E15_BM vs. E21_BM; (**B**) E15_BM vs. E27_BM; (**C**) E21_BM vs. E27_BM.

As a useful approach during the investigation of various integrated metabolic pathways, a KEGG pathway analysis of the DAMs among three comparison groups was performed; most of the DAMs were enriched in metabolic pathways, biosynthesis of secondary metabolites, biosynthesis of cofactors, protein digestion and absorption, and histidine metabolism, suggesting that these metabolic pathways may play important roles in the muscle development of duck during the embryonic stage (Figure 6). The statistics of pathway enrichment analysis for the differential metabolites was also carried out, and l-glutamic acid was the most significantly up-regulated metabolite, followed by n-acetyl-1-aspartylglutamic acid, l-2-aminoadipic acid, 3-hydroxybutyric acid, and bilirubin. In contrast, the most significantly down-regulated metabolites were palmitic acid, 4-guanidinobutanoate, myristic acid, 3-dehydroxycarnitine, and s-adenosylmethioninamine, respectively, indicating their important roles in the regulation of duck skeletal muscle development.

### 2.2. Transcriptome Profiles

#### 2.2.1. Overview of Transcriptome

To identify potential candidate genes affecting skeletal muscle in ducks during embryonic development, the gene expression profiles of breast muscles from Pekin ducks were examined using RNA-Seq. RNA was prepared from three breast muscles at different developmental stages (E15, E21 and E27), and nine cDNA libraries were then constructed. After sequencing and filtering, there were over 62 million filtered reads; the total mapped rate was 87.79~88.38%, and the uniquely mapped rate was 83.71~84.92%. All Q20 values were > 99.9%, and the Q30 value was up to 97.3% (Table 1).

#### 2.2.2. Identification of Differentially Expressed Genes

**Figure 7:**
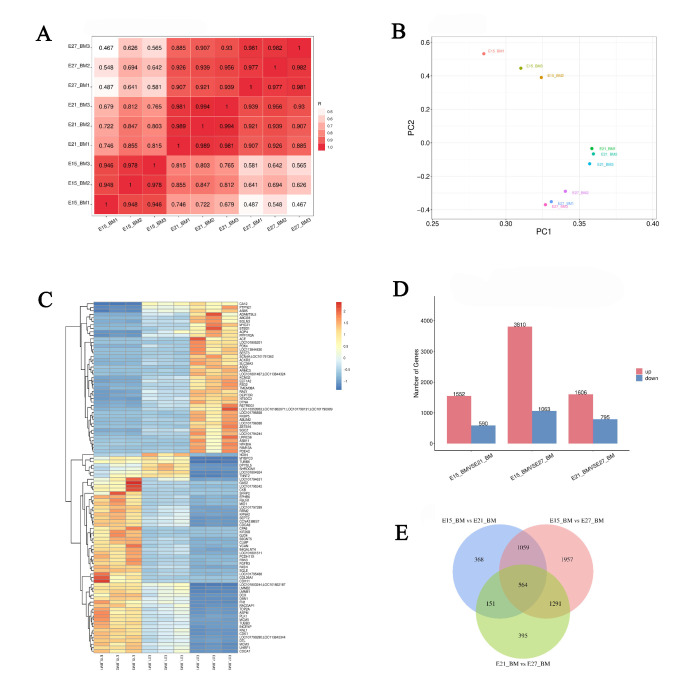
Analysis of differential gene expression. (**A**) Correlation analysis of patterns of gene expression in each group; (**B**) principal component analysis of differentially expressed genes; (**C**) cluster analysis of differentially expressed genes; (**D**) number of differentially expressed genes in each group; (**E**) Venn diagram of co-expression DEGs.

The correlation analysis based on the gene expression profiles revealed that the correlations among two samples per group both were greater than 0.94, which indicated that the biological replicates were reliable in this study (Figure 7A). In concert with this, the principal component analysis (PCA) showed a distinguishable distribution in each group, and gene expression clusters of one group were clearly departed from the other two groups (Figure 7B). Similar results were also found in the heat map, that is, the expression of genes in each sample group was different (Figure 7C). A total of 13,383 DEGs were found in the three comparison groups (E15_BM vs. E21_BM, E15_BM vs. E27_BM and E21_BM vs. E27_BM). Among 2142 DEGs in the breast muscle of Pekin duck from E15_BM vs. E21_BM group, 1552 were up-regulated genes and 590 were down-regulated genes, while among 4873 DEGs in E15 vs. E27 group, the number of up-regulated genes was 3810 and the down-regulated genes was 1063. In addition, there were 2401 DEGs from E21_BM vs. E27_BM, including 1606 up-regulated genes and 795 down-regulated genes (Figure 7D). In breast muscle of Pekin duck, 564 DEGs were co-expressed across three developmental time points during the embryonic stage (Figure 7E). The top ten up- and down-regulated genes in each comparison group were listed in Table 2.

#### 2.2.3. Go Ontology and KEGG Pathway Analysis

**Figure 8:**
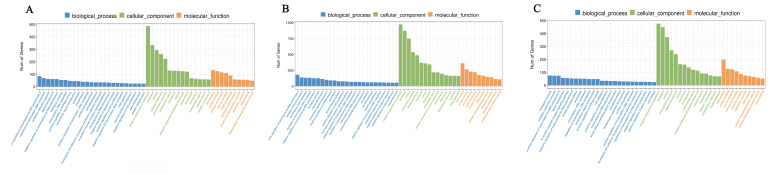
GO enrichment analysis of differentially expressed genes (DEGs) in breast muscle. (**A**) E15_BM vs. E21_BM; (**B**) E15_BM vs. E27_BM; (**C**) E21_BM vs. E27_BM.

These DEGs were categorized into three main GO categories including biological process, cellular component, and molecular function. In total, there were 456, 508, and 435 significantly enriched GO terms (*p* < 0.05) identified in E15_BM vs. E21_BM, E15_BM vs. E27_BM, and E21_BM vs. E27_BM, respectively. The significantly enriched terms from biological process in the comparisons were associated with muscle or cell growth and development, such as positive regulation of cell proliferation, regulation of cell cycle, actin filament organization, and regulation of actin cytoskeleton organization (Figure 8, Table 3). One hundred and thirty DEGs among the three comparison groups were associated with these terms, with some of these genes having been reported to be associated with growth, such as *MYOG*, *SMYD1*, *MYH9*, *FGF10*, *TNNT2*, *IGFBP3*, *MYOD1*, and *MEF2C*.

**Figure 9:**
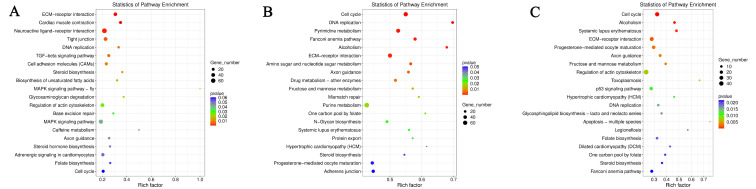
KEGG pathways of differentially expressed genes (DEGs) enrichment in breast muscle. (**A**) E15_BM vs. E21_BM; (**B**) E15_BM vs. E27_BM; (**C**) E21_BM vs. E27_BM.

Then, the DEGs were annotated using KEGG to identify enriched pathways, and seven significantly enriched pathways related to growth and development of breast muscle were identified, including focal adhesion, regulation of actin cytoskeleton, wnt signaling pathway, insulin signaling pathway, extracellular matrix (ECM)-receptor interaction, cell cycle, and adherens junction (Table 4). Eleven genes, *FYN*, *PTK2*, *PXN*, *CRK*, *CRKL*, *PAK*, *RHOA*, *ROCK*, *INSR*, *PDPK1*, and *ARHGEF*, were highly enriched in GO terms and significantly up-regulated or down-regulated expressed in KEGG pathways to regulate the development of skeletal muscle in Pekin duck during the embryonic stage (Figure 9).

### 2.3. Integrated Analysis of Transcriptomics and Metabolomics

**Figure 10:**
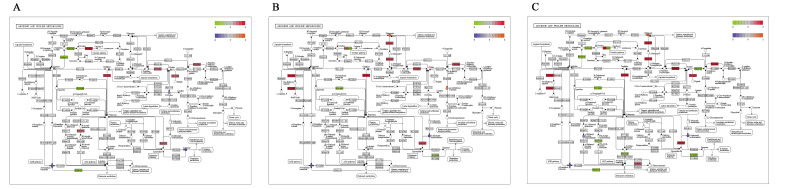
The pathway of Arginine and proline metabolism. (**A**) E15_BM vs. E21_BM; (**B**) E21_BM vs. E27_BM; (**C**) E15_BM vs. E27_BM.

**Figure 11:**
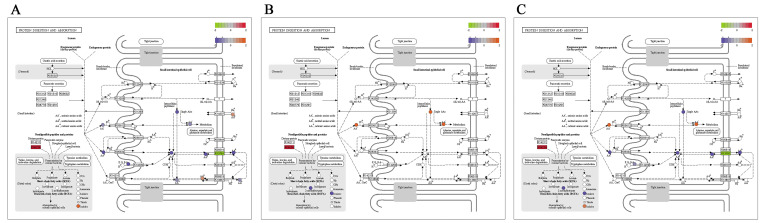
The pathway of protein digestion and absorption. (**A**) E15_BM vs. E21_BM; (**B**) E21_BM vs. E27_BM; (**C**) E15_BM vs. E27_BM.

**Figure 12:**
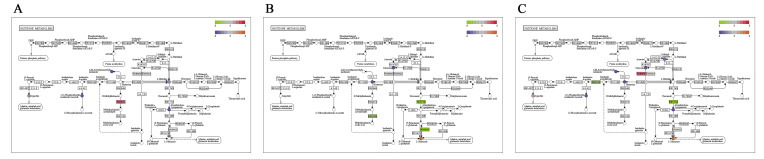
The pathway of histidine metabolism. (**A**) E15_BM vs. E21_BM; (**B**) E21_BM vs. E27_BM; (**C**) E15_BM vs. E27_BM.

The KEGG pathway of metabolomics and transcriptomics were integrated and analyzed. By interactively comparing the metabolomic and transcriptomic data, the potential metabolites and the corresponding differentially expressed genes at the molecular and biochemical levels were identified, and 25 pathways were found in 3 comparison groups (Table S3). It is noteworthy that pathways involved in arginine and proline metabolism, protein digestion and absorption, and histidine metabolism were enriched significantly in both transcriptomic and metabolomic data (Figure 10, Figure 11 and Figure 12), which will provide a theoretical basis for revealing the genetic regulation mechanism of duck muscle development. 

### 2.4. RT-qPCR Validation of the RNA-Seq Data

**Figure 13:**
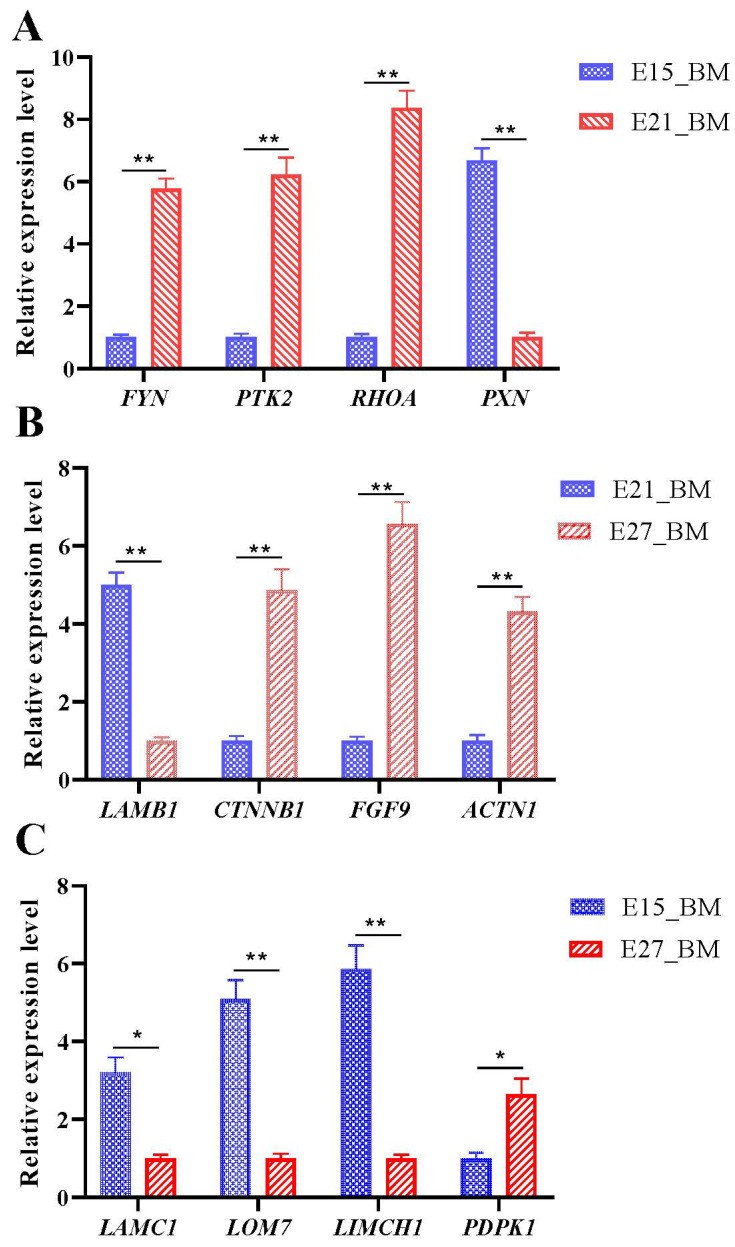
Validation of differentially expressed genes. (**A**) E15_BM vs. E21_BM; (**B**) E21_BM vs. E27_BM; (**C**) E15_BM vs. E27_BM. “*” was considered significant difference (*p* < 0.05); “**” was considered extremely significant difference (*p* < 0.01).

Twelve DEGs from the six significantly enriched pathways (related to growth and development of breast muscle) were randomly selected to validate the results of RNA-Seq. RT-qPCR was performed on the same RNA samples as used for RNA-Seq. The results showed a similar regulated trend in the expression of these genes, which confirmed the validity of the data from RNA-Seq (Figure 13).

## 3. Discussion

Meat quality is the key factor to determine the economic value of modern animal husbandry, and it is also an important reference index for duck breeding. Skeletal muscle development is regulated by DEGs, and the concentration and proportion of endogenous metabolites change with the extension of development time, showing certain differences. The identification of key genes and metabolites for skeletal muscle development in embryonic duck is helpful to explain the molecular mechanism of poultry muscle development. Moreover, The DAMs and DEGs can be used as molecular markers and candidate genes to provide data support for breeding new duck varieties (strains) based on molecular breeding methods, and also provide a method reference for future research on the genetic mechanism of important economic traits in livestock and poultry. Due to the limited evidence provided by single omics and the existence of ambiguous data, the problem cannot be fully explained. Therefore, the combination analysis of multi-omics has been widely used [19,20]. Here, the transcriptomic and metabolomic changes of muscle development in Pekin duck during the embryonic stage were tracked, which can provide a unique opportunity for us to deeply understand the candidate genes and metabolites in the process of avian skeletal muscle development. It has been known that primary myofibers form about E6 of incubation, and secondary muscle fibers begin to differentiate between E12 and E16. Some studies have shown that myofiber size in breast muscle of duck embryos decreased by 55% from E22 to hatching (d 28), which is the occurrence of muscle fiber atrophy [6]. Therefore, from the middle stage of incubation, we collected samples every six days until the day before hatching, that is, the breast muscles at 15, 21, and 27 d of embryonic stage were selected for sequencing analysis. E15 was chosen because it is the time of secondary muscle fiber formation and also the middle stage of duck embryo incubation. E21 was selected because it was the period when the muscle fiber atrophy did not occur in the duck embryo, while E27 was the last period of duck embryo muscle fiber atrophy.

### 3.1. Metabolome Analysis

In the present study, the pathways which participate in metabolic pathways, biosynthesis of secondary metabolites, biosynthesis of cofactors, protein digestion and absorption, and histidine metabolism may contribute to the dynamic process of skeletal muscle development in embryonic duck. Specifically, metabolic pathway refers to a series of continuous metabolic reactions, leading to the synthesis or decomposition of certain metabolites, in which decomposition mainly completes the work of obtaining energy and the “raw material” required for body composition, while synthesis mainly accomplishes the utilization of energy storage and the “raw material” to construct the components of the organism [21]. Likewise, protein digestion and absorption are the basic organic matter that constitute cells. It is essential to renew or repair tissues and maintain the nutritional balance in the body [22]. Histidine is a semi-essential amino acid, which is particularly important for the growth of infants and animals, and histidine metabolism also plays an important role in biological metabolism during development [23]. Moreover, a secondary metabolite is a kind of non-essential small-molecule organic compound produced by secondary metabolism for cell life activities or normal biological growth and development [24], and cofactors refer to non-protein compounds that bind to enzymes and are necessary for catalyzing reactions [25]. The biosynthesis of the two is important for the cell and tissue development of organisms.

Metabolites are the key regulators and markers of animal growth and development. In short, the metabonomic analysis results of this study strongly suggest that the accumulation of these important metabolites of glutamate, n-acetyl-1-aspartylglutamic acid, l-2-aminoadipic acid, 3-hydroxybutyric acid, and bilirubin may directly contribute to promoting the development of embryonic duck skeletal muscle. Glutamate plays a key role in all transamination reactions in the body and in many other metabolic pathways in different organs (including skeletal muscle) [26], and n-acetyl-1-aspartylglutamic acid also affects muscle development [27]. Sato et al. showed that l-2-aminoadipic acid regulates protein turnover of C2C12 myotube [28], and 3-hydroxybutyric acid possesses a key role in promoting muscle development and maintaining muscle protein balance [29]. Similarly, bilirubin has a cytoprotective effect on various oxidative damages, and it can reduce ectopic lipid deposition in skeletal muscle and liver cells, which is the main key factor in the pathogenesis of diabetes mellitus type 2 [30].

Beyond that, there are some metabolites down-regulated during the development of skeletal muscle in the duck embryo, which also play an important role. Palmitic acid regulates muscle development by negatively affecting myotube diameter, fusion, and metabolism [31]. 4-guanidinobutanoate participates in a variety of metabolic activities and has high physiological activity [32]. Myristic acid can not only improve the secretion of insulin, but also improve the sensitivity of tissues to insulin action [33]. In addition, researchers have shown that both 3-dehydroxycarnitine and s-adenosylmethioninamine have unique metabolic characteristics related to the muscle or cell [34,35].

Therefore, these metabolic pathways and metabolites identified in this study are crucial for the development of skeletal muscle in embryonic duck.

### 3.2. Transcriptome Profiles

During muscle development, myoblasts go through the steps of migration, adhesion, elongation, intercellular recognition, alignment, and myoblastic membrane fusion, and finally form myotubes. With the development of high-throughput sequencing, screening genes related to muscle development has attracted increasing attention, and massive amounts of transcriptional data have been produced. In this study, KEGG analysis showed that the DEGs were assigned to more than 17 significant pathways, and the identified genes were mainly enriched in focal adhesion, regulation of actin cytoskeleton, wnt signaling pathway, insulin signaling pathway, ECM-receptor interaction, cell cycle, and adherens junction.

The site where integrin and proteoglycan mediated adhesion connects with the actin cytoskeleton is called focal adhesion (FA), which is dynamic multi-protein complexes that connect ECM with the intracellular cytoskeleton [36,37]. The formation and maturation of FA is a key procedure during myoblast differentiation. FAK (focal adhesion kinase), a non-receptor tyrosine kinase, plays a key role in the reorganization of the sarcomere by acting as a scaffold for the recruitment of focal adhesion protein [38], and as part of the mechanism related to the load and fiber type of fully developed muscle tissue, FAK regulates the dynamic formation and turnover of FAs and the molecules that control myofibrillar protein synthesis [39]. Moreover, FAK can also regulate cell proliferation and migration by recruiting additional kinases and inducing complex signal cascades [40]. The interaction between cells and ECM is the key to regulating cell and tissue homeostasis. ECM consists of a complex mixture of structural and functional macromolecules, mainly including collagen, fibronectin, and laminin [41]. Multiprotein complexes, such as FAs and fibrous adhesions, mediate cell-matrix adhesion, connect the ECM to the cytoskeleton, and promote cell-mediated matrix remodeling. ECM not only provides substrates for cell adhesion, but also influences various cellular functions, including cell survival, proliferation, migration, and differentiation, through signaling proteins located in and near the adhesion complex [42,43]. Studies have shown that cell differentiation was directly or indirectly controlled by the interaction between cells and ECM proteins [44]. Actin is a well-known cytoskeletal protein, and the actin cytoskeleton plays a role in development and reproduction [45]. Studies have indicated that cytoskeleton formation participates in cell migration and differentiation [46,47], and actin is closely related to contraction and myoblast differentiation [48]. Many morphological changes in cells are driven at least in part by the remodeling of the actin cytoskeleton [49].

Repetition of the cell cycle orchestrates genome duplication and the subsequent segregation of each genome duplication into new daughter cells, resulting in the proliferation of cells [50]. Cell proliferation and differentiation are closely related to signal pathways that regulate the cell cycle-controlled gene expression during animal development [51]. The regulation of the cell cycle is important not only for cell differentiation during development, but also for morphogenesis [52]. Cells are connected together by cell–cell junctions (adherens junctions, tight junctions, and desmosomes), which are critical to the homeostasis of tissues, especially during embryonic development and tissue maintenance when cells are constantly squeezed and stretched [53,54]. Adherens junction is well-known cell–cell junction structure, and is the attachment site of cadherin adhesion receptor to link the actin cytoskeleton of adjacent cells [55]. It is the site of mechanosensing and signal transmission, and regulates the dynamics of actomyosin, which then generates the force driving morphogenesis [56].

Wnt pathway, also known as the β-catenin pathway, plays a crucial role (multiple developmental events) during embryogenesis in vertebrates, and regulates the homeostatic processes in adulthood [57]. It can enhance the proliferation and differentiation of embryonic cells and other metabolic functions, and is an important regulator of the total number of muscle cells and the ratio of fast muscle cells to slow muscle cells [58,59]. In addition, the pathway also regulates cytoskeletal rearrangement during embryonic development [60], and lipid metabolism, glucose homeostasis, and energy balance [61]. Lu et al. found that the polymorphism of Wnt signaling pathway genes (*RHOA*, *Wnt3A*, *CHP*, *RAC1*, *Wnt1*, *Wnt9A*, *MAPK9*) was significantly related to chicken carcass traits, indicating that the Wnt signaling pathway played a major role in regulating chicken production traits (carcass characteristics) [62]. Insulin facilitates the entry of glucose into fat and muscle, where it is stored as intracellular triglycerides and glycogen [63], and 75% of insulin mediated glucose uptake and utilization is carried out by skeletal muscle, indicating that the development of skeletal muscle is closely related to insulin [64,65]. Rhoads et al. found that insulin played an important role in skeletal muscle growth by regulating muscle hypertrophy, protein accumulation, and cell activity [66]. The insulin signaling pathway is a mechanism that regulates growth rate in response to nutrient availability [67]. Ma et al. transplanted hSKM (human skeletal myoblasts) into mouse skeletal muscle, and the transcription of multiple genes related to the insulin signaling pathway, and mitochondrial biogenesis and function were altered [68].

Briefly, cytoskeleton formation and ECM-integrin receptor interactions participate in myoblast differentiation [69]. Specifically, myoblast migration depends on the dynamics of the cytoskeleton, which is mainly related to actin and regulatory factors, and elongation, adhesion, and intercellular recognition in myoblasts mainly rely on the interactions between integrins and cytoskeletal proteins. Moreover, the ECM was connected to the actin cytoskeleton by FA [70,71,72]. Likewise, wnt signaling pathway, insulin signaling pathway, cell cycle, and adherens junction have been suggested to regulate the progressions of skeletal muscle transcription, cell proliferation, and differentiation during embryonic development. These pathways found in this study, including FA, regulation of actin cytoskeleton, wnt signaling pathway, insulin signaling pathway, ECM-receptor interaction, cell cycle, and adherens junction, synergistically promote skeletal muscle development in embryonic Pekin ducks.

In this study, 11 genes (*FYN*, *PTK2*, *PXN*, *CRK*, *CRKL*, *PAK*, *RHOA*, *ROCK*, *INSR*, *PDPK1*, and *ARHGEF*) were also identified, which were likely to be involved in duck muscle development and were either up- or down-regulated with more than several-fold changes.

*FYN* is a member of the Src kinase family with diverse biological functions, including regulation of mitogenic signaling and proliferation and integrin-mediated interactions, as well as cellular growth, survival, adhesion, motility, T-cell receptor signaling, and cytoskeletal remodeling [73]. Yamada et al. have shown that Fyn/STAT3/Vps34 signaling pathway can regulate fiber-type specific macroautophagy and muscle atrophy of mouse skeletal muscle [74]. *PTK2* (protein tyrosine kinase-2) encodes FAK, and controls its expression. There is an association between muscle specific force and *PTK2* SNPs [75,76]. *PXN* (paxillin) has two homologues, *PXN*-*1* and *PXN*-*2*. *PXN*-*1* may play a regulatory role in the matrix of *C. elegans*, and *PXN*-*2* is very important for the later steps of the formation of the basement membrane, especially when mechanical adhesion is required between the tissue and the ECM. Lee et al. suggested that *PXN*-*1* may play a role in the attachment of tissues and the guidance of neurons during the development of *C. elegans* [77]. Gotenstein et al. showed that *PXN-2* was crucial to the embryogenesis of the *C. elegans* and the inhibition of the regeneration of adult axons [78].

*CRK*, including two splicing variants *Crk* I and *Crk* II, regulates signal transduction processes involving growth regulation, cell transformation, cell migration, and cell adhesion [79]. *Crk* II can be localized with other FA related proteins, such as Src, FAK, and paxillin [80]. Likewise, *CRKL* (Crk-like gene) participates in many signaling pathways and controls cell morphology, cell movement, cell proliferation, and differentiation [81]. There is a compelling evidence that Crk and Crk-like (Crkl) physically interacting with Dock proteins are required for myoblast fusion in zebrafish [82]. *PAK* (P21-activaed kinase), a serine/theronine kinase, is important for participants in insulin signaling and glucose homeostasis in muscle, pancreas, liver, and other tissues [83]. It is involved in cell proliferation, apoptosis, metastasis, and cytoskeleton remodeling. Varshney and Dey et al. showed that *PAK2* can regulate glucose uptake and insulin sensitivity of neuronal cells [84]. *PDPK1* (3-phosphoinositol dependent protein kinase-1) is a member of AGC serine/threonine kinase family, and the PDPK1/AGC kinase signaling pathway is involved in the regulation of cell proliferation, growth, autophagy, and apoptosis related physiological processes [85], as well as promotion of muscle growth [86]. *PDPK-1* is closely related to the insulin signaling pathway, which can stimulate the increased catalytic activity of PDPK-1 in a PI3K-dependent manner [87].

*RHOA*, one of the Rho subfamily members of small GTPases, regulates cell proliferation and motility, and is considered to be the key regulator of actin cytoskeleton dynamics and organization in most cell types [88]. Likewise, *ROCK* (Rho-associated kinase) can regulate the contraction of stress fibers by regulating the phosphorylation level of myosin light chain [89]. Wozniak et al. believed that the contraction of stress fibers mediated by Rho and *ROCK* could regulate the formation of FA in vivo, which may regulate downstream signaling pathways and cell behavior [38]. Studies have shown that *RhoA* and *Rock* play a unique and independent regulatory role in the process of myogenic differentiation [90]. Likewise, RhoA/ROCK signaling in skeletal muscle also plays an important role [91]. *ARHGEF3*, also called *XPLN*, a RhoA/B-specific GEF, can be used as the effect of RhoGEF on RhoA/B, or the inhibitor of mTORC2-Akt signaling, negatively regulating myoblastic differentiation [92]. Moreover, *ARHGEF3* may also control skeletal muscle regeneration and strength through autophagy presenting in the ARHGEF3 RhoA/B-ROCK signaling pathway [93]. *INSR* (insulin receptor) is a central starting point of insulin signaling, and is involved in the glucose homeostasis mechanism, proliferation, and growth of skeletal muscle and fat cells [94]. Interestingly, muscle differentiation is blocked by RhoA/Rho kinase through serine phosphorylation of insulin receptor substrates-1 and -2 [95]. 

These pathways and genes play an important role and may be potential markers in duck skeletal muscle development, but their mechanisms need further experimental verification. 

### 3.3. Integrated Analysis of Transcriptomics and Metabolomics

Through the combined analysis of transcriptome and metabolome, 26 significantly enriched pathways were identified that may regulate the muscle development of Pekin duck during the embryonic stage. Among these pathways significantly enriched, three pathways including arginine and proline metabolism, protein digestion and absorption, and histidine metabolism were found to be commonly enriched with DEGs (*CKB*, *AGMAT*, *SRM*, *ODC1*, *GATM*, *P4HA2*, *MYADML2*, *GAMT*, *NOS2*, *PYCR3*, *LAP3*, *SMOX*, *P4HA3*, *ALDH18A1*, *COL19A1* and *UROC1*) and DEMs (4-guanidinobutanoate, l-glutamic acid, histamine, l-isoleucine, l-aspartic acid, 4-imidazolone-5-propanoate, s-adenosylmethioninamine, indole, 2-methylbutyric acid, 1-(5-phosphoribosyl)imidazole-4-acetate, 4-(beta-acetylaminoethyl)imidazole and piperidine). 

It is well known that amino acids play a role in muscle growth; for example, arginine can promote skeletal muscle fiber type transformation from fast-twitch to slow-twitch via Sirt1/AMPK pathway [96], and proline can increase the rates of protein synthesis in the muscle [97]. Histidine is also required for skeletal muscle development [98]. Amino acid metabolism is mainly used to synthesize proteins, polypeptides, and other nitrogenous substances needed by the body, and can also be converted into sugars, lipids, or can re-synthesize some non-essential amino acids. It can also be oxidized into carbon dioxide and water through the circulation of tricarboxylic acid and release energy. Similarly, under the action of protease, protein is eventually decomposed into amino acids and finally absorbed by the intestine through metabolism.

## 4. Materials and Methods

### 4.1. Animals and Sample Collection

A total of 120 eggs of Pekin duck were incubated in a standard incubator according to the conventional incubation procedure. Eighteen embryos were randomly picked out from day 15 (E15), day 21 (E21), and day 27 (E27) of the incubation period, and the breast muscles were collected and immediately frozen in liquid nitrogen for RNA and DNA extraction. DNA was extracted according to the phenol-chloroform protocol, and sex identification primers (gCHD, F: 5′TGCAGAAGCA ATATTACAAGT3′; R: 5′AATTCATTATCATCTGGTGG3′) [99] were used to determine the sex of embryos. Because the vast majority of duck farms are laying ducks, and the same gender can also avoid the error of sequencing data, female embryos in this study were selected as the research objects. Animal care, slaughter, and experimental procedures were approved by Institutional Animal Care and Institutional Ethic Committee of Northwest A&F University (ethic code: #1201/2021).

### 4.2. Extraction of Metabolites and Metabolomics Analysis

#### 4.2.1. Metabolites Extraction

Six breast muscle samples of female embryos at each embryonic stage were randomly selected for the extraction of metabolites. The 100 mg samples were ground with liquid nitrogen and extracted with 120 μL of precooled 50% methanol. Then, the mixtures were vortexed and mixed well, and incubated at room temperature for 10 min. The extractions were stored overnight at −20 °C to precipitate the protein in the samples. After centrifugation at 4000× *g* for 20 min, the supernatants were transferred into new 96-well plates. Metabolic samples were stored at −80 °C prior to LC-MS analysis, and the quality control (QC) sample was prepared by mixing an equal aliquot of the supernatants (10 μL) from all of the samples.

#### 4.2.2. LC-MS/MS Analysis

All chromatographic separations were performed using a Thermo Scientific UltiMate 3000 HPLC (Thermo Scientific, Waltham, MA, USA), equipped with an ACQUITY UPLC BEH C18 column (100 × 2.1 mm, 1.8 µm, Waters, UK) for the reversed phase separation. The auto-sampler temperature was 4 °C, and the flow rate was 0.4 mL/min. The mobile phase consisted of solvent A (water, 0.1% formic acid) and solvent B (Acetonitrile, 0.1% formic acid). The analysis was carried out with elution gradient as follows: 0~0.5 min, 5% B; 0.5~7 min, 5% to 100% B; 7~8 min, 100% B; 8~8.1 min, 100% to 5% B; 8.1~10 min, 5% B. The injection volume for each sample was 4 µL.

The metabolites eluted form the column were detected by high-resolution tandem mass spectrometer Q-Exactive (Thermo Scientific, Waltham, MA, USA). The Q-Exactive was operated in both positive and negative ion modes. Precursor spectra (70~1050 m/z) were collected at 70,000 resolution to hit an AGC target of 3e6. The maximum inject time was set to 100 ms. A top 3 configuration to acquire data was set in DDA mode. Fragment spectra were collected at 17,500 resolution to hit an AGC target of 1e5 with a maximum inject time of 80 ms. In order to evaluate the stability of the LC-MS during the whole acquisition, a quality control sample (pool of all samples) was acquired after every 10 samples.

#### 4.2.3. Processing and Analysis of Metabolome Data

The raw data were transformed into a readable data format (mzXML) using MSConvert software (GUI) of Proteowizard. The peak extraction and quality control were performed using XCMS software and the extracted substances were annotated with ions using CAMERA software. The metabolite identification was carried out using metaX software (primary mass spectrometry information for database matching identification, and secondary mass spectrometry information for matching identification with in-house standard database). Multivariate statistical analysis was applied, including unsupervised principal component analysis (PCA) and supervised partial least square discriminant analysis (PLS-DA). Moreover, univariate analysis was performed, including student’s *t*-test and fold change analysis. The metabolites with a variable importance in projection (VIP) values ≥ 1 and *p*-value < 0.05 were used as criteria to discover differentially expressed metabolites (DEMs). Metabolites identifications were carried out based on the metabolites information public database, such as HMDB and mzCloud, and analyses of metabolic pathways were conducted using the Kyoto Encyclopedia of Genes and Genomes (KEGG, http://www.kegg.jp/kegg, accessed on 30 November 2022).

### 4.3. RNA Sequencing and Data Analysis

#### 4.3.1. RNA Extraction and Library Preparation

Total RNA was extracted from breast muscle tissues of 3 female embryos from each embryonic stage (randomly selected from the same samples used for metabolome sequencing) using Trizol reagent (Invitrogen, Carlsbad, CA, USA) following the manufacturer’s protocol. The RNA purity and concentration were verified by agarose gel electrophoresis and Nanodrop 2000 (Thermo, Waltham, CA, USA). The sample integrities were determined based on Agilent 2100 Bioanalyzer (Agilent Technologies, San Jose, CA, USA) to ensure that the RNA integrity number (RIN) was above 8.0. Nine sequencing libraries were constructed, and their quality was tested. The paired-end sequencing was performed on an Illumina Hiseq 4000 (LC Bio, Hangzhou, China) following the vendor’s recommended protocol, and a paired end read of 150 bp was generated.

#### 4.3.2. RNA Sequencing and Data Analysis

After sequencing, clean reads were obtained by removing reads containing adapters or poly-N, more than 10% of unknown nucleotides and low-quality reads containing more than 50% of low-quality (Q-value ≤ 10) bases from raw reads. At the same time, quality parameters for filtered data including Q30, GC content, and sequence-duplication level were used for data filtering. All the downstream analyses were based on clean reads with high quality. These clean reads were then mapped to the *Anas platyrhynchos* genome sequence (https://www.ncbi.nlm.nih.gov/genome/?term=DUCK, accessed on 30 November 2022) and annotated transcripts (https://www.ncbi.nlm.nih.gov/assembly/GCF_003850225.1, accessed on 30 November 2022). Only data with perfect match reads, or one mismatch were further analyzed and annotated based on the reference genome. The Hisat2 tool software were used to map with the reference genome.

To compare the expression profiles in different samples, the gene expression levels were normalized by fragments per kilobase per million fragments (FPKM). FPKM represents the number of sequencing fragments contained in each thousand transcriptional sequencing bases per million sequenced bases. Differential expression was analyzed using the edgeR software (v.3.20, accessed on 4 December 2022), and the false discovery rate (FDR) <0.01 and fold change ≥2 were set as the threshold for screening differentially expressed genes (DEGs). Gene Ontology (GO) enrichment analysis was implemented by the GOseq R software package (https://bioconductor.org/packages/release/bioc/html/goseq.html, accessed on 4 December 2022), and Kyoto Encyclopedia of Genes and Genomes (KEGG) pathway analysis and functional annotation for DEGs were performed using KOBAS 3.0.

#### 4.3.3. Validation of RNA-seq Data

In order to confirm the reliability of data, eighteen DEGs were selected to validate the RNA-Seq results. The cDNAs were synthesized from the same RNA samples used for transcriptome sequencing according to the manual of reverse transcription kit (TaKaRa, Dalian, China). The β-actin was used as an internal control, and the list of primers were described in Table 5. The RT-qPCR were performed using TransStart Tip Green qPCR SuperMix (Transgen, Beijing, China) on a EcoRT48 (OSA, London, UK). Three technical replicates were carried out per sample. The relative gene expression levels were calculated using the 2^−ΔΔCt^ method and the results were expressed as mean ± SD of at least three independent biological replicates. The difference was analyzed using one-way analysis of variance (ANOVA) followed by Dunnet’s *t*-test and Tukey’s test, and *p* < 0.05 was considered significant difference and *p* < 0.01 was considered extremely significant difference.

### 4.4. Integrative Analysis of Metabolomics and Transcriptomics

For the integrated analyses of transcriptome and metabolome, all DEGs and DAMs were simultaneously mapped into the KEGG database to obtain their common pathway information, and clarify the main biochemical pathways and signal transduction pathways DEGs and DEMs are involved in.

## 5. Conclusions

In summary, many candidate genes and metabolites involved in crucial biological pathways underlying muscle development in Pekin duck during the embryonic stage were successfully identified according to transcriptomic and metabolomic datasets in this study. DAMs in the metabolome were significantly enriched in metabolic pathways, biosynthesis of secondary metabolites, biosynthesis of cofactors, protein digestion and absorption, and histidine metabolism in three comparison groups. DEGs, including *FYN*, *PTK2*, *PXN*, *CRK*, *CRKL*, *PAK*, *RHOA*, *ROCK*, *INSR*, *PDPK1,* and *ARHGEF*, were mainly enriched in focal adhesion, regulation of actin cytoskeleton, wnt signaling pathway, insulin signaling pathway, ECM-receptor interaction, cell cycle, and adherens junction. Moreover, by interactively comparing metabolomic and transcriptomic data, DEGs (*CKB*, *AGMAT*, *SRM*, *ODC1*, *GATM*, *P4HA2*, *MYADML2*, *GAMT*, *NOS2*, *PYCR3*, *LAP3*, *SMOX*, *P4HA3*, *ALDH18A1*, *COL19A1,* and *UROC1*) were highly correlated with the corresponding metabolites (4-guanidinobutanoate, l-glutamic acid, histamine, l-isoleucine, l-aspartic acid, 4-imidazolone-5-propanoate, s-adenosylmethioninamine, indole, 2-methylbutyric acid, 1-(5-phosphoribosyl)imidazole-4-acetate, 4-(beta-acetylaminoethyl)imidazole, and piperidine) that were involved in arginine and proline metabolism, protein digestion and absorption, and histidine metabolism. These results will provide basic materials for further investigating the molecular mechanism of skeletal muscle development in duck.

## Figures and Tables

**Table 1:** RNA-Seq data from breast muscle of Pekin duck.

Sample Name	Raw Data	Clean Data	Q20 %	Q30 %	GC Content%	Mapped Reads	Unique Mapped Reads	Multi Mapped Reads
E15_BM1	77,109,784	70,263,996	99.97	97.81	45.50	62,101,245 (88.38%)	59,533,956 (84.73%)	2,567,289 (3.65%)
E15_BM2	74,321,278	67,100,234	99.97	97.78	45.50	59,133,997 (88.13%)	56,909,871 (84.81%)	2,224,126 (3.31%)
E15_BM3	77,985,728	70,738,438	99.97	97.72	45.50	62,172,644 (87.89%)	59,569,330 (84.21%)	2,603,314 (3.68%)
E21_BM1	77,175,666	71,023,652	99.97	97.74	46.50	62,546,291 (88.06%)	59,905,449 (84.35%)	2,640,842 (3.72%)
E21_BM2	76,696,598	70,756,258	99.97	97.82	45.50	62,300,708 (88.05%)	59,702,570 (84.38%)	2,598,138 (3.67%)
E21_BM3	76,451,028	69,392,604	99.97	97.67	45.50	61,290,597 (88.32%)	58,817,525 (84.76%)	2,473,072 (3.56%)
E27_BM1	72,954,478	66,093,658	99.97	97.73	44.50	58,411,962 (88.38%)	56,127,311 (84.92%)	2,284,651 (3.46%)
E27_BM2	70,316,234	62,796,684	99.96	97.79	46.50	55,131,121 (87.79%)	52,566,867 (83.71%)	2,564,254 (4.08%)
E27_BM3	75,114,974	67,389,576	99.97	97.35	44.50	59,482,822 (88.27%)	57,015,130 (84.61%)	2,467,692 (3.66%)

Note: E15_BM, breast muscle of Pekin duck on day 15 of the incubation period. The same is shown below.

**Table 2:** Top 10 up- and down-regulated genes in each comparison group.

Comparison Group	Up-Regulated Genes	Down-Regulated Genes
E15_BM vs. E21_BM	*CLEC3A*, *TCF21*, *NKX2*-*1*, *LOC101799083*, *RXFP2*, *NRN1*, *IFITM10*, *LOC101789807*, *LOC101800713*, *GDF5*	*LOC113842946*, *LOC113844954*, *LOC101792564*, *CA4*, *LOC101803753*, *NKX2-2*, *LOC110353429*, *LOC113842952*, *LOC113842945*, *LOC101795455*
E15_BM vs. E27_BM	*ADRB3*, *NKX2*-*8*, *WNT9B*, *TMPRSS2*, *ZIC3*, *SERPINB5*, *PENK*, *LOC101805062*, *ANXA10*, *LOC113841609*	*CA4*, *TRNAH*-*GUG*, *LOC113844954*, *LOC101793166*, *MLN*, *LOC101805201*, *LOC101793608*, *IL4I1*, *LOC101800226*, *SYCP2L*
E21_BM vs. E27_BM	*LOC101795455*, *LOC106016676*, *LOC101804301*, *LOC101798615*, *LOC101794100*, *HEMGN*, *LOC101791390*, *SIAH3*, *MNX1*, *BD2*	*CLEC3A*, *NPVF*, *TP53TG5*, *TRNAH-GUG*, *MLN*, *HS3ST5*, *PTCHD4*, *LOC113844884*, *LOC113844846*, *PTH*

**Table 3:** GO terms related to the processes of muscle or cell growth and development.

ID	GO Terms	Number of DEGs	*p*-Value
GO:0008284	positive regulation of cell proliferation	113	0.0009
GO:0008285	negative regulation of cell proliferation	98	0.0049
GO:0008283	cell proliferation	85	0.0030
GO:0006457	actin cytoskeleton organization	52	0.0084
GO:0007409	regulation of cell cycle	44	0.0065
GO:0090305	actin filament organization	28	0.0066
GO:0045454	positive regulation of cell growth	27	0.0266
GO:0050919	regulation of actin cytoskeleton organization	19	0.0010
GO:0000278	embryo development	16	0.0003
GO:0043044	skeletal muscle contraction	12	0.0285
GO:0047496	negative regulation of myoblast differentiation	9	0.0164
GO:0016051	myoblast differentiation	9	0.0164
GO:0001953	muscle cell differentiation	8	0.0059
GO:0060394	positive regulation of myotube differentiation	7	0.0484
GO:0030048	striated muscle contraction	7	0.0484
GO:0045820	regulation of skeletal muscle cell differentiation	6	0.0213
GO:0021707	embryonic body morphogenesis	5	0.0405
GO:0051415	striated muscle tissue development	5	0.0405
GO:0051224	skeletal muscle thin filament assembly	5	0.0405

**Table 4:** KEGG pathways related to growth and development of breast muscle.

ID	Pathway Name	Number of DEGs	*p*-Value
ko04510	Focal adhesion	143	0.004168
ko04810	Regulation of actin cytoskeleton	133	0.001607
ko04310	Wnt signaling pathway	104	0.002853
ko04110	Cell cycle	85	0.000004
ko04910	Insulin signaling pathway	81	0.021021
ko04512	ECM-receptor interaction	77	0.007448
ko04520	Adherens junction	53	0.009627

**Table 5:** Primers used for RT-qPCR validation.

Name	Sequences (5′-3′)	Accession No.	Length	Regulated
*FYN*	F: TTGCTGCCGCTTAGTAGTCC	XM_027454688.2	200 bp	Up
	R: TGCCAGGCTTCAGAGTCTTG			
*PTK2*	F: AATCCAGGCGACAAGTCACG	XM_038175670.1	178 bp	Up
	R: ATCCCGTGAGAACCAGGGTA			
*RHOA*	F: ACGAGCACACAAGACGAGAG	NM_001310346.1	198 bp	Up
	R: ACCCGGACTTTTTCTTGCCA			
*PXN*	F: TGAATCGGGACCTCTCTCCA	XM_038187676.1	203 bp	Down
	R: ACCATTCTGCTCCTCTCCCT			
*LAMB1*	F: AAGAAACGCTGAACAACGCC	XM_038175878.1	172 bp	Down
	R: TTCAGCTCGGTGTTGAGGAC			
*CTNNB1*	F: ATACCGCCCAGACGATCCTA	XM_027450106.2	138 bp	Up
	R: GGCAGACCATCAACTGGGTA			
*FGF9*	F: TCTGATGGCTCCCTTAGGTG	XM_005031210.5	127 bp	Up
	R: CTCAGACTGACCCAGGTGGT			
*ACTN1*	F: AGCAGACCAACGACTACATGC	XM_038179254.1	125 bp	Up
	R: AGCCTTGCGGAGGTGAGAAT			
*LAMC1*	F: CTTCAACCTCCAGAGCGGAC	XM_038182826.1	165 bp	Down
	R: TCAGGGCCAAATCCGAAGTG			
*LMO7*	F: CTTCCTCTGCCAAAGCACCT	XM_038183971.1	138 bp	Down
	R: TCTTCCCACTGACTGACCTG			
*LIMCH1*	F: ATCGAGCAAGTGACAGGCAG	XM_027457183.2	138 bp	Down
	R: AATGGGGGTCGGTAGTCTGT			
*PDPK1*	F: GAGCTACGTCCAGAAGCCAA	XM_027469114.2	127 bp	Up
	R: GCCGCCACACTTCATGTATC			
*β-actin*	F: CCCTGTATGCCTCTGGTCG	EF667345	194 bp	
	R: CTCGGCTGTGGTGGTGAAG			

## Data Availability

The datasets generated for this study can be found in the NCBI SRA (Submission: SUB12535066). Bioproject #PRJNA923756 and Biosamples #SAMN32740501-SAMN32740509.

## Supplementary Materials

The supporting information can be downloaded at https://www.mdpi.com/article/10.3390/ijms24065214/s1.
