# Supplementary material for: Integration of Transcriptomics and Non-Targeted Metabolomics Reveals the Underlying Mechanism of Skeletal Muscle Development in Duck during Embryonic Stage
Source: Int J Mol Sci. 2023 Mar 8;24(6):5214. doi: 10.3390/ijms24065214 (PMC10049352; doi:10.3390/ijms24065214)
Supplement: Supplementary file 1 [file ijms-24-05214-s001.zip › Figure S1.pdf]

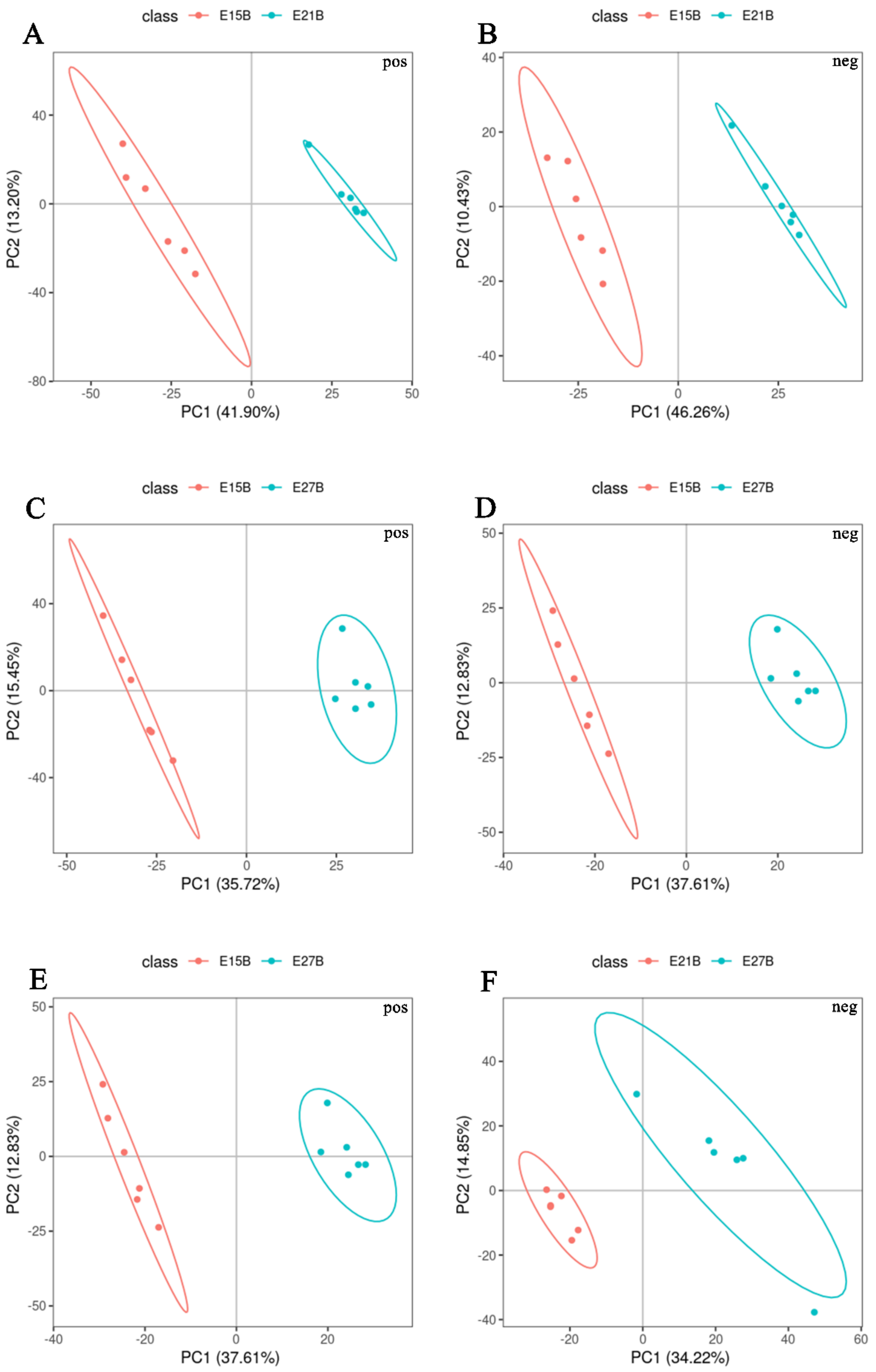

Figure S1: Overall differences of the three comparison groups in the positive and negative ion modes.
