# Supplementary material for: Integration of Transcriptomics and Non-Targeted Metabolomics Reveals the Underlying Mechanism of Skeletal Muscle Development in Duck during Embryonic Stage
Source: Int J Mol Sci. 2023 Mar 8;24(6):5214. doi: 10.3390/ijms24065214 (PMC10049352; doi:10.3390/ijms24065214)
Supplement: Supplementary file 1 [file ijms-24-05214-s001.zip › Table S1.pdf]

**Table S1.** Number of differentially accumulated metabolites (DAMs) in positive ion mode (pos) and negative ion mode (neg) of three comparison groups

| Comparison group | All DAMs (pos) | Up DAMs (pos) | Down DAMs (pos) | All DAMs (neg) | Up DAMs (neg) | Down DAMs (neg) |
|---|---|---|---|---|---|---|
| E15_BM vs E21_BM | 599 | 243 | 356 | 399 | 183 | 216 |
| E15_BM vs E27_BM | 584 | 219 | 365 | 349 | 135 | 214 |
| E21_BM vs E27_BM | 388 | 156 | 232 | 299 | 134 | 165 |
