# Supplementary material for: Integration of Transcriptomics and Non-Targeted Metabolomics Reveals the Underlying Mechanism of Skeletal Muscle Development in Duck during Embryonic Stage
Source: Int J Mol Sci. 2023 Mar 8;24(6):5214. doi: 10.3390/ijms24065214 (PMC10049352; doi:10.3390/ijms24065214)
Supplement: Supplementary file 1 [file ijms-24-05214-s001.zip › Table S2.pdf]

Table S2. Top 10 up- and down-regulated DAMs in each comparison group.

Comparison group	Up- regulated DAMs	Down-regulated DAMs
E15_BM vs E21_BM	Caprolactam, biliverdin, (2e)-2-tridecene-4,6,8-triyn-1-ol, carbofuran, acetamiprid, 1-phenylpropane-1_2-dione, methyl alpha-aspartylphenylalaninate, aspartame, 4,4′ -dicarboxy-2,2′ -bipyridine, d(+)-phenyllactic acid	2,4-quinolinediol, n-[4-(diethylamino)phenyl]-n'-phenylurea, 5-[5-hydroxy-3-(hydroxymethyl)pentyl]-8a-(hydroxymethyl)-5,6-dimethyl-3,4,4a,5,6,7,8,8a-octahydronaphthalene-1-carboxylic acid, 2-cyclohexyl-4,6-dinitrophenol, prostaglandin f2α, 3-methyldioxyindole, ent-prostaglandin f2α, (±)17(18)-epete, limaprost, 1a,1b-dihomo prostaglandin f2α
E15_BM vs E27_BM	bis(methylbenzylidene)sorbitol, (2z)-3-(3,4,5-trimethoxyphenyl)-2-propen-1-yl acetate, 2-ethoxy-2-oxoethyl ethyl phthalate, caprolactam, biliverdin, 2-hydroxy-3-{[5-hydroxy-3-(4-hydroxyphenyl)-4-oxo-4h-chromen-7-yl]oxy}succinic acid, hypoglycin a, (2e)-2-tridecene-4,6,8-triyn-1-ol, o-[{(2r)-3-[(4z,7z,10z,13z,16z,19z)-4,7,10,13,16,19-docosahexaenoyloxy]-2-hydroxypropoxy}(hydroxy)phosphoryl]-l-serine, 1-(4-amino-4-carboxybutanoyl)-2-piperidinecarboxylic acid	2,4-quinolinediol, carnosine, 1a,1b-dihomo prostaglandin f2α, ent-prostaglandin f2α, prostaglandin f2α, 5-[5-hydroxy-3-(hydroxymethyl)pentyl]-8a-(hydroxymethyl)-5,6-dimethyl-3,4,4a,5,6,7,8,8a-octahydronaphthalene-1-carboxylic acid, (±)17(18)-epete, 2-cyclohexyl-4,6-dinitrophenol, 3-methyldioxyindole, (+/-)8-hepe
E21_BM vs E27_BM	r(+)-iaa-94, glu-asp, fludarabine phosphate, sulfoxone, 3-dehydrocarnitine, ethyl 3-oxo-3-(2,4,5-trifluorophenyl)propanoate, cidofovir anhydrous, temurtide, l-glutamic acid, dinoseb	carnosine, gabazine free base, (1s_4s)-4-hydroxy-3-oxocyclohexane-1-carboxylate, mutagen x, 4-(5-chloro-2h-benzotriazol-2-yl)-3-hydroxyphenyl benzoate, thiamine, motesanib, n-(indol-3-ylacetyl)glutamine, carnosine(beta-alanyl-l-histidine), 2,3-dihydro-1-benzofuran-2-carboxylic acid
