# Supplementary material for: Integration of Transcriptomics and Non-Targeted Metabolomics Reveals the Underlying Mechanism of Skeletal Muscle Development in Duck during Embryonic Stage
Source: Int J Mol Sci. 2023 Mar 8;24(6):5214. doi: 10.3390/ijms24065214 (PMC10049352; doi:10.3390/ijms24065214)
Supplement: Supplementary file 1 [file ijms-24-05214-s001.zip › Table S3.pdf]

Table S3. The KEGG pathways of metabolomics and transcriptomics in 3 comparison groups.

KEGG	Pathway
ko00240	Pyrimidine metabolism
ko04068	FoxO signaling pathway
ko04080	Neuroactive ligand-receptor interaction
ko00330	metabolism
ko00630	Glyoxylate and dicarboxylate metabolism
ko04974	Protein digestion and absorption
ko04024	cAMP signaling pathway
ko00220	Arginine biosynthesis
ko00250	Alanine, aspartate and glutamate metabolism
ko00072	Synthesis and degradation of ketone bodies
ko00230	Purine metabolism
ko00061	Fatty acid biosynthesis
ko02010	ABC transporters
ko00380	Tryptophan metabolism
ko00480	Glutathione metabolism
ko00340	Histidine metabolism
ko01040	Biosynthesis of unsaturated fatty acids
ko00970	Aminoacyl-tRNA biosynthesis
ko00360	Phenylalanine metabolism
ko00410	beta-Alanine metabolism
ko00062	Fatty acid elongation
ko00650	Butanoate metabolism
ko00053	Ascorbate and aldarate metabolism
ko00270	Cysteine and methionine metabolism
ko00071	Fatty acid degradation
ko00860	Porphyrin and chlorophyll metabolism
